# Bridging Research and Policy on Climate Change and Conflict

**DOI:** 10.1007/s40641-018-0119-9

**Published:** 2018-10-27

**Authors:** Elisabeth A. Gilmore, Lauren Herzer Risi, Elizabeth Tennant, Halvard Buhaug

**Affiliations:** 1 Department of International Development, Community and Environment, Clark University, 950 Main St., Worcester, MA 01610 USA; 2 Peace Research Institute Oslo, Oslo, Norway; 3 Environmental Change and Security Program, Wilson Center, Washington, DC 20004-3027 USA

**Keywords:** Policy, Research agenda, Climate change, Armed conflict, Development

## Abstract

**Purpose of Review:**

This special issue on “Bridging Research and Policy on Climate Change and Conflict” brings together the results of a 2018 workshop organized by the Peace Research Institute, Oslo (PRIO) and the Wilson Center with six papers that address different aspects of the translation of the research on climate change and conflict to policy and practice. Here, we provide an overview of the workshop and papers to highlight key opportunities and challenges to linking the climate-conflict scholarship with pressing issues in diplomacy, development, and security.

**Recent Findings:**

Multiple methods, especially comparative case studies, should be applied to elucidate the more complex mechanisms of the climate-conflict link. This approach may also enhance engagement with the policymakers who draw on examples and narratives. There is also a need for both predictive models that capture contextual factors and policy interactions as well as decision-support tools, such as integrated assessment models, that can be used to test the implications of different theories and models in the literature.

**Summary:**

Scholars should engage the policy community to formulate research questions that are more policy relevant, such as the effectiveness of interventions. There is also the need for models and frameworks that help practitioners synthesize the academic results. Practitioners are encouraged to leverage the comparative advantages of academic researchers in new policy and projects to inform data collection and future analysis of effectiveness.

## Introduction

The past few years have seen both an increase in violence as well as an increase in extreme weather events, prompting a continued policy discourse about the potential associations between climate change and security risks. Since 2013, there has been the largest increase in violence and armed conflict since the termination of the Cold War, although there is still evidence of longer term declines in terms of conflict lethality [1, 2]. At the same time, while attribution of any specific weather event to climate change remains challenging, we are observing more events that are consistent with the predicted impacts of climate change [3]. Even if it is possible to implement stringent climate policies that would limit end of century global warming to 1.5 °C, some effects of climate change, such as sea-level rise, may be irreversible [4] and the burden of these impacts may be highly unequal [5, 6]. While there are still important questions about whether, how, and when the physical impacts of climate change may be implicated in armed conflict and violence [see recent papers such as [7, 8]] as well as possibilities for cooperation [see recent papers such as [9, 10]], the potential extent of the climate impacts on many aspects of human wellbeing continues to prompt both scholarship and policy. As a result, researchers and practitioners have made substantial progress in our understanding of how climatic changes may alter or enhance the propensity for new violence or interact with existing conflicts. More tightly coupling the efforts of the research community with the needs and experience of policymakers could further enhance the development of evidence-based policy to more rapidly and effectively address the challenges presented by climate change and conflict.

In this introduction to the 2018 special issue on “Bridging Research and Policy on Climate Change and Conflict”, we highlight a recent workshop hosted by the Peace Research Institute Oslo (PRIO) and the Wilson Center on fostering dialog between the research and decision-making communities. We then provide an overview of the six papers in this issue that were invited following this meeting that look at different aspects of research and policy integration for climate change and conflict. We conclude with recommendations to enhance the dialog between the research community and the security, development and diplomacy practitioners, and policy-makers.

## Fostering Dialog Between Research and Policy on Climate Change and Conflict

In March 2018, the Peace Research Institute Oslo (PRIO) and the Wilson Center co-hosted a workshop for a small group of researchers, policymakers, and policy influencers drawn from academia, the United States (US) government, international agencies, and NGOs. Participants discussed the state of the research on climate change and conflict, the relevance of scholarship to the pressing policy challenges in diplomacy, development, and security, and how to enhance the integration between research and practice. At the workshop, the participants were asked to consider the following two questions:

1. What are the challenges towards integrating the ‘state of the science’ research into policy?
2. How can we improve the dialog and feedback between these two communities?

To focus attention on the key issues, we invited three experts to present on topics of high relevance to understanding and managing climate change and conflict, but where divergences and gaps between the scholarship and policy exist. The first presentation by Katharine J. Mach (Stanford University) showed how to employ expert elicitation and scenarios to gain insight into the degree of scientific consensus around the causal links and pathways, the magnitude of the effect of climate change on conflict for different underlying conditions, and the uncertainty in these estimates [see [11] for methods]. Second, Nina von Uexkull (Uppsala University & Peace Research Institute Oslo) reviewed the state of the literature on the relationship between agriculture, livelihood, and instability, highlighting her work showing that even if climate change cannot be linked to the onset of armed conflict in general, climatic effects may be especially harmful in vulnerable regions that are already experiencing conflict [see [12]]. Finally, Tor A. Benjaminsen (Norwegian University of Life Sciences & Peace Research Institute Oslo) discussed the interactions between conflicts over land in areas of unclear property rights and land-based climate mitigation policy [see [13, 14]]*.* After each presentation, the experts and the workshop participants engaged in substantive discussions about the research, the relevance of the work to policy and practice, and opportunities to translate the work into the policy space. A summary of the research presentations is available in the report of the workshop. (A report of this meeting is available at https://www.wilsoncenter.org/publication/bridging-research-and-policy-climate-change-and-conflict).

Three key recommendations emerged at the workshop on how to improve the translation of scientific research for the development of policy interventions as well as opportunities to enhance the co-production of knowledge by the policymakers and the scientific community.

1. The largely quantitative studies that underpin much of the climate-conflict literature should be coupled with examples and narratives that are more useful to policy-makers. The research community is generally divided into quantitative and qualitative methods. The quantitative work aims to identify underlying relationships and general patterns and can be interpreted as the probability that a conflict will be observed given certain conditions. Qualitative scholars also provide insight into these relationships through detailed tracing of conditions and interactions through case studies. While this information is in principle of high value to policy, these research approaches are difficult to translate for policymakers as they need to answer questions about generalizable patterns as well as counterfactuals (e.g., why some locations with observed environmental stresses also observe conflict and others do not). Narratives that compare locations with different degrees of violence, as well as efforts to ground-truth empirical findings, can help illustrate how research conclusions would be observed in a real-world context.
2. In addition to testing theories and explaining underlying relationships between climatic stressors and conflict, the research community should ask questions related to the effectiveness of policies and interventions. While there is the need to understand the causal pathways to different forms of violence, policymakers also require information on interventions related to preventing the onset of conflict, supporting the cessation of existing violence, and peacebuilding. Retrospective evaluations, including monitoring and evaluation (M&E), as well as examples of how they play out on the ground, across the basket of programs and policies implemented by development and security communities could inform future efforts. Further, the comparative advantages of researchers can be further leveraged to evaluate the effectiveness of policies and projects by informing early data collection to facilitate these analyses as well as making use of comprehensive datasets that can be generated through joint activities. There is considerable untapped potential in enhancing collaboration between scholars and practitioner communities in responding to these challenges.
3. Finally, synthesizing this knowledge into models that can provide predictive information on the onset and evolution of conflicts, as well as decision support on interventions and when to deploy them, would improve the uptake of research by decision-makers. Predictive models, such as early-warning systems, play an important role in conflict, development, and security communities. While there is a long history and some skepticism of forecasting in the conflict community [15], policymakers are looking for additional guidance for their efforts. Decision-support tools could also enhance the translation of existing and new research results to the policy community. While this class of models can have predictive capacity, they often place equal or greater emphasis on learning about the system. The integrated assessment models (IAMs), such as those that are used to evaluate the costs and effectiveness of different climate policies, and system dynamic approaches may be useful frameworks. Starting with a framework that synthesizes the existing knowledge of the dynamics of conflict and climate change, alternative descriptions of these interactions and novel findings can be implemented into the model to allow decision-makers to explore and update their understanding of the system.

## Climate Change and Conflict Research as a Critical Input for Policy Interventions

Following the workshop, members of the researcher and policy communities were invited to provide their insights. Here, we provide summaries and context for these papers, highlighting the broader themes of research and policy interactions for climate change and conflict.

The overview piece in this issue is prepared by Busby (2018), Taking Stock: The Field of Climate and Security [16]. In this essay, Busby surveys the progression and state of the science on climate and security over the past 15 years and outlines potential directions for policy-relevant research. He argues that researchers should focus their efforts on elucidating the intermediary mechanisms and contextual factors that may link climate change to conflict rather than continue to debate whether or not there are any direct associations. While efforts that focus on the causal pathways may be better matched to the needs of policymakers, Busby emphasizes that the existing academic evidence thus far is too limited and inconclusive to inform policy on specific practices, institutional arrangements, and other factors to mitigate the risk of climate-related conflict and insecurity [17]. He suggests investigating the following pathways and contextual factors: shocks to food prices or changes in agricultural production, economic mechanisms, environmentally driven migration, and the role of institutions [18]. He further stresses the potential for more complex interactions, such as disasters, which might lead to conflict via economic mechanisms or inadequate government response. He also addresses the strengths and limitations of the different methods. Statistical analysis may be useful in identifying hotspots of vulnerability and developing early warning systems similar to FEWSNET [19]. However, Busby urges scholars to apply theories and knowledge to specific cases and ideally comparative case studies to generate the kind of contextual understanding and to ask questions that address the policy-makers’ need to design effective interventions [20].

In their public statements, high-level policymakers and influencers frequently emphasize the importance and influence of a few cases that are popularly believed to be driven by or indicative of the types of conflict and security risks that may be observed due to climate change [21, 22]. Three regions that are frequently mentioned are the ongoing conflict in Syria [23, 24], the vulnerabilities in the Lake Chad Basin [25, 26], and the pastoral-herder and other conflicts in the Horn of Africa [27], although the research and policy communities do not always draw the same conclusions about the contribution of climate change relative to political and economic factors. In his paper, Ide (2018) demonstrates how the proposed links between the 2006–2009 drought in Syria and the 2011 onset of civil war in Syria exemplifies the shortcomings of the climate-conflict literature [23, 24, 28]. He reviews the evidence for the causal chain that is frequently presented in the literature: [1] climate change contributed to the severe drought, [2] this drought disrupted agriculture and livelihoods in rural areas, [3] the loss of livelihood led to large scale migration to the urban centers, and [4] the additional population strained resources in the receiving areas, prompting grievances that contributed to anti-regime protests and ultimately led to the civil war. Ide finds support for the migration into the urban centers due to the effects of the drought on agricultural incomes, but emphasizes that the magnitude of these effects relative to political and economic factors is not well understood. He finds much weaker evidence for attribution of the drought to anthropogenic climate change and for the link between migration and the onset of conflict. However, challenges with integrating quantitative and qualitative research methodologies as well as the lack of rigorous theories on how the environment influences conflict limit the cumulation of policy-relevant knowledge on the Syria case and climate-conflict linkages more broadly. Ide argues that scholars should draw on the full range of methods and theories to build general knowledge about causal and contextual factors [29], knowledge that might help policy-makers to prevent future conflict and instability.

In addition to improving the basis for how contextual factors affect the potential influence of climate change on conflict, there is also a need to understand the interactions with policy, specifically aid, development, and diplomacy. Investigating the links between disasters, such as those due to extreme weather events, and collective violence highlights both the contextual factors and the influence of a wide range of policies. In his review of recent research on disasters and violence, Brzoska (2018) finds some support for an increase in collective violence; however, the actions of individuals and policy-makers can alter not only the magnitude but also the directionality of this association [30]. First, he stresses that political factors and institutions influence not only the potential for disasters to lead to violence, but also the severity of a disaster [31]. Second, he emphasizes that the effects of weather are generally small and secondary and tend to reinforce existing conditions and processes. As such, disasters may hasten processes of peace-building and de-escalation as well as precipitating or exacerbating conflict [32]. To identify practical entry-points for reducing the incidence and duration of violent conflict precipitated by disasters, Brzoska recommends research into the pre-event conditions (e.g., livelihoods, ethnic marginalization, and institutions) and post-disaster mechanisms that may lead to conflict [33]. Possible post-disaster pathways include shifts in economic and resource constraints, grievances and perceived injustice, migration, and shocks to the resources and capabilities of collective actors and institutions.

Climate adaptation and mitigation policy also has the potential to influence the onset and incidence of conflict, specifically through altering land use [34]. While the stresses posed by the physical impacts of climate change on land use and the implications of these effects for human security and conflict are often addressed in the literature, Froese and Schilling (2018) describe a broader and more complex nexus between climate change, land use, and conflict that emphasizes the role of individual and policy responses to climate change Froese and Schilling under review. Climate change mitigation projects, such as the siting of renewable energy generation or forest conservation programs (for example REDD/REDD+) can result in changes in land use, the costs and benefits of which often accrue unequally [35, 36]. When these changes impact human security, such as livelihoods and quality-of-life, it may exacerbate existing conflicts or create new ones [37]. Froese and Schilling particularly highlight the likelihood of land use change to negatively impact already poor and marginalized populations, due in part to their weak or informal land tenure [38]. They also outline a pathway from climate adaptation to conflict, whereby actions with diffuse benefits (i.e., flood protections) may have land use impacts that disproportionately affect specific individuals or groups that may already be marginalized. Despite the gaps in the understanding of how these policies may be related to conflict, Froese and Schilling suggest a list of best practices for managing the potential for adverse effects from these policies, namely community participation, comprehensive impact analyses, and trust building. These activities may be even more valuable when implementing climate policy in situations with existing conflicts.

Institutions underlie both the potential for climate to introduce security risks and the effectiveness of policies to mitigate adverse impacts [39]. At the same time, research on the role of institutions and their responses to climate security is limited and tends to focus on global governance, such as the UN Security Council [10, 40]. Krampe and Mobjörk (2018) provide insight into how policymakers define climate security risks and the opportunities and organizational constraints for managing these risks. They conduct a comparative case study of the policy documents from security Intergovernmental Organizations (IGOs) in Asia and Africa to investigate how these organizations conceptualize and respond to climate security risks [41]. They find that while Western IGOs tend to understand climate risk in terms of state security, the organizations in developing countries frame the challenges of climate change and conflict in terms of human security, such as food access. This allows for more entry points for climate security; however, policy implementation commensurate with these risks is limited both by the issues of sovereignty and trust between states and organizational divisions within IGOs. Additionally, the parts of the organizations that are charged with managing the climate risks are often secondary compared to other functions of the IGO.

Finally, Schweizer (2018) starts a discussion on the use of decision support for conflict and climate risks through the use of scenario analysis Schweizer under review. She outlines how existing methodologies of foresight and decision support, and advances in the application of these methods to climate change problems more broadly, can be built on to learn from existing evidence and provide tools for learning and experimentation. Schweizer (2018) surveys a range of existing methods for applying foresight to decision support, and their various strengths and limitations—including knowledge requirements, transparency and potential for bias, and suitability for purpose. For example, even if the state of theory and empirical consensus is advanced, the validity of predictive exercises is likely to weaken over time as climate change shifts human and natural systems further from their historical states. While novel in the climate-conflict space [42], Schweizer (2018) discusses how the use of scenario analysis has resulted in important advances in climate change research [43, 44]. Additionally, she encourages climate-conflict researchers to engage with the scenario frameworks developed by the climate change research community: the Representative Concentration Pathways (RCPs) and the Shared Socioeconomic Pathways (SSPs) [43, 45]. Working from the RCP-SSP scenarios not only allows for an analysis of the co-occurrence of different socioeconomic and climatic conditions, but also facilitates the comparability of studies and therefore the accumulation of knowledge on climate-conflict links.

## Conclusions and Recommendations for Enhancing Policy and Research Interactions

Taken together, the workshop and the papers highlight both the progress that has been made by the research community as well as the gaps to addressing the needs of the policy community. One of the main recommendations is to take a mixed methods approach—coupling quantitative and qualitative methods—to investigate the climate–conflict relationship. From the perspective of the scholars, the expectation is that this approach will help elucidate the more complex contextual factors. Specifically, in-depth single case studies and comparative case study approaches, especially of cases without conflict where the quantitative models predict a high risk of conflict, are seen as a favorable way forward. This is consistent with the calls from the practitioners to have the quantitative efforts combined with case studies; however, the presentation of the results in narratives may be more persuasive for policymakers. Further, as Busby (2018) stresses, scholars should be encouraged to ask questions that are more consistent with those of practitioners. One way forward discussed in the workshop was to leverage the skills of scholars, involving them with the structure and data collection of interventions to facilitate independent evaluations. Further, there is a need to investigate how the more complex interactions between institutions and policies, such as climate mitigation and adaptation policies and humanitarian aid for disasters, can affect the underlying conditions on which climate may act to precipitate conflict.

The practitioners also stressed the need for more tools to aid in decision-making, asking for both predictive models as well as models for decision support. Presently, there are few examples of these types of models in the literature for climate change and conflict. Approaches to develop predictive models are presently dominated by early warning models, such as FEWSNET [19]. Newer approaches to prediction that incorporate theory are being developed that have short to medium term predictive capacity. Decision support efforts that incorporate scenario analysis are also emerging, such as those described in [46, 47]. These models are designed to provide a basis for both cumulation as well as to test alternative theories of conflict and development. There is progress on coupling of conflict models with the scenario framework used by the climate research community (e.g., the RCP-SSP scenarios). However, there is substantial work to be done so that these models can be used not only to investigate the climate change—conflict links, but also the effectiveness of different policy levers to reducing these risks.

Through the workshop and this associated special issue, we do not expect to develop a consensus on the relationships between climate and conflict nor a single path forward. The absence of consensus in the literature, however, should not be seen as a barrier to developing evidence-based policy and nor are these challenges unique to climate change and conflict. Examples of these discussions can be found in almost every discipline [48]. As developed in the National Research Council report (2012), the process by which policy is developed and implemented often involves arguments and reasoning that “differ[s] from and can contradict scientific reasons”. The evidence from scholars needs to be positioned within this context. While few scholars maintain that there is a direct association between climate change and conflict, there are still many open questions about the pathways from climate impacts to conflict. Policymakers may draw upon this evidence and may also be influenced by the dialog on security issues and the experiences from a broader range of actors. While it is not the role of academics to provide operational guidance, developing a greater understanding of pressing policy needs can frame what questions are asked, how the research is conducted, and to whom the results are communicated. The Wilson Center workshop and this special issue constitute a step towards continued dialog on how academic work can be linked to on the ground practices. Such efforts can lead to the development of a base of actionable science for policy on climate change and conflict.
